# Simulation and analysis of lead-free perovskite solar cells incorporating cerium oxide as electron transporting layer

**DOI:** 10.1039/d2ra05957f

**Published:** 2022-11-11

**Authors:** Ali K. Al-Mousoi, Mustafa K. A. Mohammed, Rahul Pandey, Jaya Madan, Davoud Dastan, G. Ravi, P. Sakthivel, G. Anandha babu

**Affiliations:** Department of Radiology and Ultrasonography Techniques, College of Medical Techniques, Al-Farahidi University 10011 Baghdad Iraq; Radiological Techniques Department, Al-Mustaqbal University College 51001 Hillah Babylon Iraq mustafa_kareem97@yahoo.com; VLSI Centre of Excellence, Chitkara University Institute of Engineering and Technology, Chitkara University 140417 Rajpura Punjab India; Department of Materials Science and Engineering, Cornell University Ithaca NY 14850 USA; Department of Physics, Alagappa University Karaikudi 630003 Tamil Nadu India; Department of Physics, Bannari Amman Institute of Technology Tamil Nadu India

## Abstract

The great demand for renewable energy has greatly contributed to the development of the solar cell industry. Recently, silicon solar cells have dominated the world market. The ease of processing gives perovskite solar cells (PSCs) an advantage over conventional silicon solar cells. Regular silicon photovoltaics require expensive, multi-step processes accomplished in a specialized ultraclean-chamber facility at an elevated temperature (>1000 °C) and highly vacuumed workspace. Hence, researchers and the solar cell industry have focused on PSC as a great rival to silicon solar cells. Despite this, the highest efficiency was obtained from lead-based PSC, which has a considerably high toxicity issue and low stability related to lead content, so the research field pays attention to lead-free perovskite solar cells. In this digital simulation, tin-based perovskite in this paper, methylammonium tin iodide (MASnI_3_) with the use of cerium oxide (CeO_*x*_) as an electron transporting layer (ETL) with varying percentages of oxygen, which means different shallow donor densities (ND). The optimum value for the thickness of the absorber layer (perovskite) was made, and the current–voltage characteristics and efficiency calculations were also accomplished for the best cell. Then an improvement was made by changing the ND value of CeO_*x*_, and the best-optimized cell parameters were: open circuit voltage (*V*_OC_) of 0.92 V, short circuit current density (*J*_SC_) of 30.79 mA cm^−2^, power conversion efficiency (PCE) of 17.77%, and fill factor (FF) of 62.86%.

## Abstract 2

In this digital simulation, tin-based with the use of CeO_*x*_ as an ETL with varying percentages of oxygen has been investigated.

## Graphical abstract

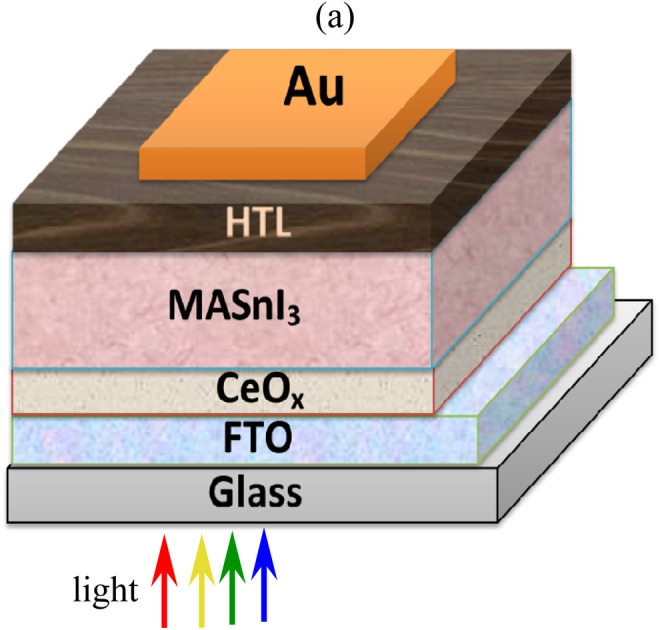

## 1. Introduction

The production of electricity is crucial for world development and is unquestionably the main driver of economic growth in both industrialized and emerging economies. A huge increase in energy demand is being fueled by both a high population growth rate and increased per capita energy consumption.^1–4^ Fossil fuel-based energy sources currently meet the majority of the world's energy needs. However, fossil fuel supplies are being depleted more quickly as energy use rises. Renewable energy solutions must be created in order to address these concerns and the growing need for energy. Solar energy, photovoltaic cells that can be used to convert directly into electricity, is the most abundant renewable energy source.^5–11^

Perovskite has gained popularity as a light-harvesting material for photovoltaic applications. Perovskite has a distinct set of optoelectrical properties, including adjustable band gaps, a high absorption coefficient, long carrier diffusion lengths, and high charge carrier mobilities.^12–16^ In 2009, Kojima and colleagues reported a power conversion efficiency (PCE) of 3.8% for perovskite solar cells (PSC).^17^ After a decade, Cui *et al.* reported a 20.8% PCE for methylammonium lead iodide PSC.^18^ CITY U HK/UW established the most recent approved efficiency of close to 25.8% in 2021.^19^ Regardless of the rapid evolution of PCE, the present stability of solar cells makes mass production of PSC difficult.^20–22^

The lead content of lead perovskite solar cells is one of their main disadvantages.^23^ Due to lead's toxicity, the Restriction of Hazardous Substances directive of the European Union forbids its use in any electronics or electrical equipment. Alternatives to lead as the metal cation in the perovskite photo-absorber have thus become a significant area of research.^24^ Stepping up or down within group IV of the periodic table of elements provides an easy way to replace lead. The recently found element, scarcely radioactively stable flerovium, lies in the row below lead. Due to its radioactivity, it may not be a suitable replacement for lead.^25^

Due to its lower toxicity, tin (Sn), which is on the same row as lead, would be an excellent replacement for lead in PSCs. Organic tin halide synthesis has been going on for as long as lead halide synthesis.^26^ The MASnI_3_ perovskite, with an energy gap (*E*_g_) of 1.3 eV, has been reported to be used in solar cells and produced a PCE of 6.4%. The +2 oxidation state of Sn, which is necessary for the formation of a perovskite, is unstable, and the metal quickly oxidizes to the +4 state when exposed to oxygen or air humidity,^27,28^ this affects both the conditions under which the device operates and the technique used to make solar cells. It has only been capable of determining the cell performance of the pure tin halide PSCs with strong sealing of the devices because any interaction with oxygen may instantaneously cause the oxidation of tin.^29^

CeO_*x*_ is regarded as one of the most significant infrequent oxides because of its wide bandgap, high dielectric constant, strong ionic conductivity, high thermal and chemical stability, matching lattice parameters with silicon, and exceptional capacity to store and release oxygen.^30,31^ Wang *et al.* prepared CeO_*x*_ (*x* = 1.87) films by a facile sol–gel approach at a low temperature (150 °C) and used them as an alternative to the high-temperature annealing processed TiO_2_ ETL. The optimized PSC achieved a champion PCE of 14.32% by adjusting the CeO_*x*_ precursor solution.^32^ Yang *et al.* used CeO_*x*_ as ETL in an inverted structure of PSC, utilizing CsPbIBr_2_ perovskite as light harvesting material. The all-inorganic PSC recorded a maximum efficiency of 5.6% with improved stability.^33^ Jien *et al.* reported CeO_*x*_ film as a protection layer for perovskite against humidity and metal reactions with the electrode. The device with CeO_*x*_ as the ETL had a PCE of 17.47%.^34^

In this simulation, CeO_*x*_ is used as an ETL material due to its tunable broad band gap (3.0–3.6 eV) and outstanding optical and dielectric properties.^35,36^ Moreover, CeO_*x*_ may enhance the stability of the PSC against moisture and oxygen.^37^ A numerical simulation of the solar cell would be necessary to figure out the best set of parameters and the physical parameters for prediction accuracy. The device is simulated using the SCAPS-1 D software and the influence of different properties of the MASnI_3_ material and CeO_*x*_ layer on the efficiency of a tin-PSC is estimated. A unique device structure (fluoride tin oxide (FTO)/CeO_*x*_/MASnI_3_/2,2′,7,7′-tetrakis[*N*,*N*-di(4-ethoxyphenyl)amino]-9,9′-spirobifluorene (Spiro-MeTAD)/Au) of a tin-based PSC using the Spiro-OMeTAD as a hole transporting layer (HTL) has been suggested for modeling. The aim of this simulation is to illustrate that the efficiency of lead-free PSCs may be enhanced by varying the ND value of the CeO_*x*_ and the thickness of the absorber layer (MASnI_3_).

## 2. Architecture and materials properties of suggested PCS

For PV modelling, a one-dimensional SCAPS simulation software was employed. The SCPAS program employs Poisson's equation, which defines the relationship between the photocarrier and the semiconductor's electrostatic potential, and continuity equations, which represent charge generation and recombination kinetics in materials.^38^ Solving both Poisson's equation and the continuity equation gives us the QE and *J*–*V* properties.^39^ Using Poisson's equation and the continuity equation, it is possible to figure out the density of electrons and holes.^40^ Poisson's equation can be used to determine the distribution of the electric field *E*(*x*):
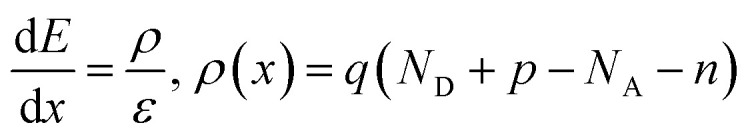


Drift and diffusion current densities control the transportation properties of charge carriers in semiconductor. The following equations describe the drift and diffusion current densities for electrons and holes.^38^
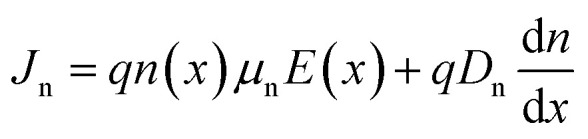

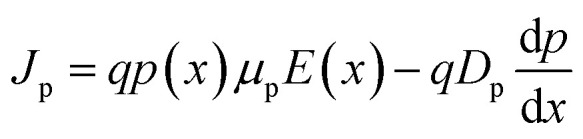
where *μ*_n_ and *μ*_p_ are to electron hole mobility, respectively, and *D*_n_, and *D*_p_ are electron and hole diffusion coefficients respectively. *ρ*(*x*), *ε*, and *q* refer to space charge distribution, dielectric permittivity, and charge of electron, respectively. *n*(*x*) and *J*_n_ represent the concentration and current density of electrons. *p*(*x*) and *J*_p_ represent the concentration and current density of hole. Seven layers, consisting of perovskite absorber, ETL, HTL, and electrodes, can be constructed into a HPSC using the SCAPS software. All PV computations in this study are performed under AM 1.5G (100 mW cm^−2^) conditions.

**Fig. 1:**
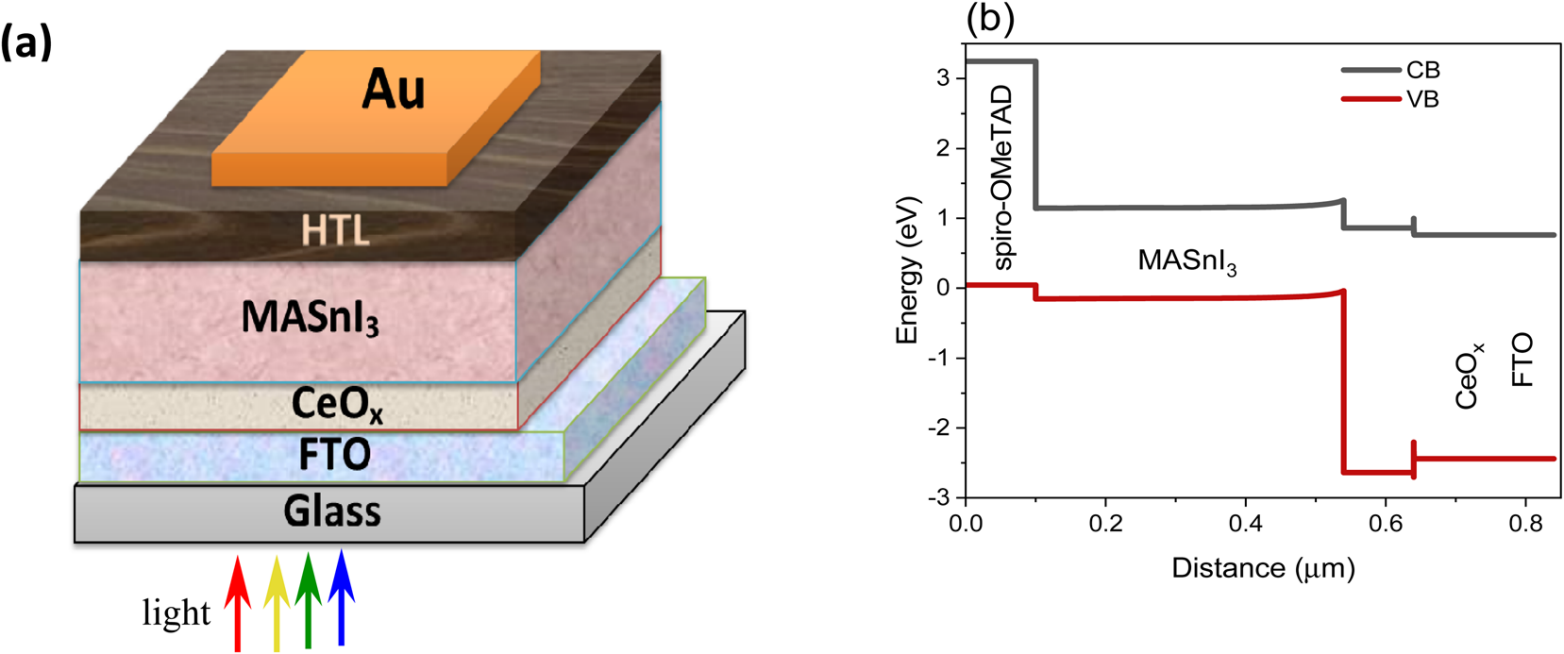
(a) Schematic layout of the designed PSC and (b) energy bands graph of the PSC.

The structure for the proposed PSC, FTO/CeO_*x*_/MASnI_3_/Spiro-OMeTAD/Au, is shown in Fig. 1a. The FTO layer, which is deposited on a glass substrate, acts as a front transparent contact. The CeO_*x*_ layer represents the crucial layer that determines the performance of the device since the transport of electrons is a major factor affecting the PCE. MASnI_3_ perovskite is the absorber material that is responsible for charge carrier generation through light absorption. Spiro-OMeTAD material acts as HTL. The energy band graph of the suggested structure (Fig. 1b) clearly shows that the conduction bands of the absorbing material MASnI_3_ are smaller than those of electron transporting material CeO_*x*_, and the mismatch between the conduction band (CB) of MASnI_3_ and the CeO_*x*_ material is very slight. As a direct consequence, electrons can easily pass from MASnI_3_ to FTO *via* CeO_*x*_. Electrons can thus move freely through CeO_*x*_ from MASnI_3_ to FTO. A very large valence band (VB) offset (VBO) exists between the absorbing material MASnI_3_ and the ETL material CeO_*x*_. Therefore, the positive charge (h^+^) in the ETL material CeO_*x*_ will be sealed. As shown in Fig. 1b, the valence bands of the hole transporting material Spiro-OMeTAD are greater than those of the absorbing material MASnI_3_, and the valence band mismatch among those two materials is significantly small. Furthermore, the offsetting in the conduction bands between the HTL material Spiro-OMeTAD and the absorbing material MASnI_3_ is very considerable, forbidding the electrons from MASnI_3_ from reaching the back contact.

The physical parameters for the simulations of the FTO/CeO_*x*_/MASnI_3_/Spiro-OMeTAD/Au heterostructure solar cell are listed in Table 1,^41^ whereas the physical properties of the defects density in MASnI_3_ are listed in Table 2. All simulations are achieved at a temperature of 300 K.

**Table 1:** Physical parameters of FTO/CeO_*x*_/MASnI_3_/Spiro-OMeTAD/Au device

Material	Spiro-OMeTAD	MASnI_3_	CeO_*x*_	FTO
Thickness of layer (μm)	0.1	0.44	0.1	0.2
Energy band gab (eV)	3.2	1.3	3.5	3.2
Electron affinity (eV)	2.1	4.2	4.6	4.4
Relative permittivity	3	10	9	9
CB effective densities of states (cm^−3^)	2.50 × 10^18^	1 × 10^18^	1 × 10^20^	2.2 × 10^18^
VB effective densities of states (cm^−3^)	1.8 × 10^19^	1 × 10^18^	2 × 10^21^	1.8 × 10^19^
Electron thermal velocity (cm s^−1^)	1 × 10^7^	1 × 10^7^	1 × 10^7^	1 × 10^7^
Hole thermal velocity (cm s^−1^)	1 × 10^7^	1 × 10^7^	1 × 10^7^	1 × 10^7^
Mobility of electron (cm^2^ V^−1^ s^−1^)	2 × 10^4^	1.6	100	20
Mobility of hole (cm^2^ V^−1^ s^−1^)	2 × 10^4^	1.6	25	10
Uniform donor densities ND (cm^−3^)	—	—	1 × 10^21^	1 × 10^21^
Uniform acceptor densities NA (cm^−3^)	1 × 10^20^	3.20 × 10^15^	—	—
Defect	1 × 10^14^	4.5 × 10^16^	1 × 10^15^	1 × 10^15^

**Table 2:** Interface parameters of FTO/CeO_*x*_/MASnI_3_/Spiro-OMeTAD/Au PSC

Parameters	Spiro-OMeTAD/MASnI_3_ interface	CeO_*x*_/MASnI_3_ interface
Defects type	Neutral	Neutral
Capture cross-section for electron (cm^2^)	1 × 10^−19^	1 × 10^−19^
Capture cross-section for hole (cm^2^)	1 × 10^−19^	1 × 10^−19^
Energetic distributions	Single	Single
Defects energy level *E*_t_	Up the maximum *E*_v_	Up the maximum *E*_v_
Reference for defect energy level *E*_t_	0.06	0.06
Total density (integrated over all energies) (cm^−2^)	1 × 10^10^	1 × 10^10^

## 3. Results and discussion

### 3.1 Optimization of MASnI_3_ thickness

**Fig. 2:**
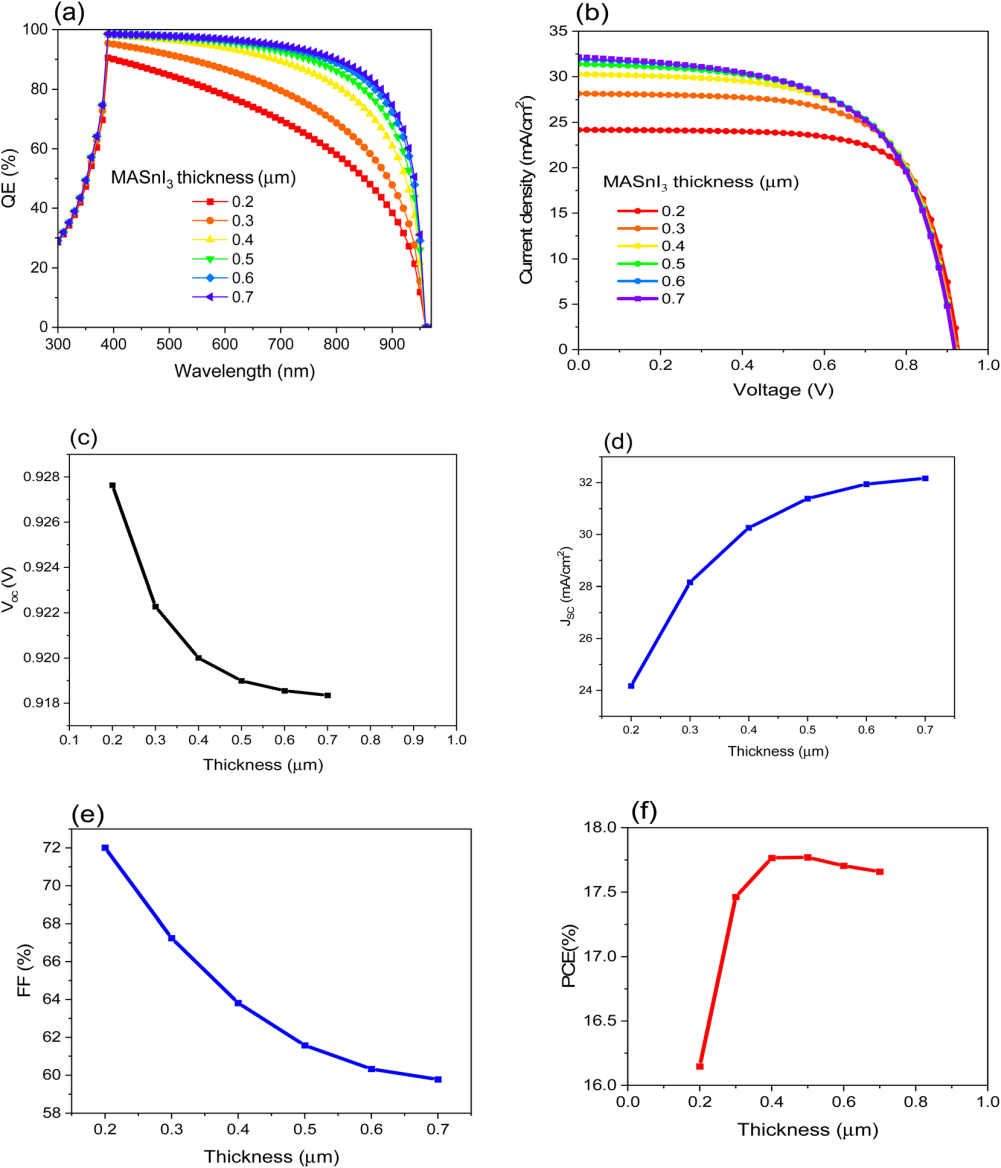
. Displays the impact of the MASnI_3_ thickness on the efficiency of FTO/CeO_*x*_/MASnI_3_/Spiro-OMeTAD/Au PSC where (a) QE, (b) *J*–*V* curve, (c) *V*_OC_, (d) *J*_SC_, (e) FF and (f) PCE.

The thickness of the absorbing material plays a significant role in solar cell efficiency since it is related to the absorption of the incident light, which leads to enhancing the *I*–*V* characteristics.^42–44^ In this study, all the parameters of the materials in Tables 1 and 2 are kept fixed, and different values of the thickness of MASnI_3_ are selected in order to estimate the optimum thickness value of the absorber material. Fig. 2 shows the parameters of the suggested PSC as a function of MASnI_3_ thickness. As Fig. 2a illustrates the quantum efficiency of the suggested PSC, we can see the influence of the absorbing material thickness on the QE value. As expected, by increasing the value of the thickness, the QE value increases. Fig. 2b demonstrates the *J*–*V* curve of the PSC under the illumination of 100 mW cm^−2^ (air mass AM 1.5G). Fig. 2c–f shows the *V*_OC_, *J*_SC_, FF, and, PCE respectively. From Fig. 2b, the *V*_OC_ is slightly decreased with thickness increases, while Fig. 2c shows a significant change in the value of *J*_SC_. Besides the variant *J*_SC_ with the thickness of the absorbing material, the FF is also highly affected by the thickness, as shown in Fig. 2d. As a consequence of the change in the last mentioned parameters, the PCE varies with changing the thickness, as illustrated in Fig. 2e. Table 3 shows the calculated parameters that are used to select the optimum thickness.

**Table 3:** Parameters of FTO/CeO_*x*_/MASnI_3_/Spiro-OMeTAD/Au PSC with different thickness of MASnI_3_

MASnI_3_ thickness (μm)	*V*_OC_ (V)	*J*_SC_ (mA cm^−2^)	FF (%)	PCE (%)	*V*_m_ (V)	*J*_m_ (mA cm^−2^)
0.2	0.928	24.172	72.010	16.147	0.754	21.406
0.3	0.922	28.158	67.243	17.463	0.726	24.056
0.4	0.920	30.260	63.815	17.766	0.711	24.993
0.5	0.919	31.386	61.573	17.770	0.704	25.217
0.6	0.919	31.948	60.327	17.703	0.702	25.218
0.7	0.918	32.169	59.776	17.659	0.701	25.185

### 3.2 Optimization of donor density (ND) of the CeO_*x*_

**Fig. 3:**
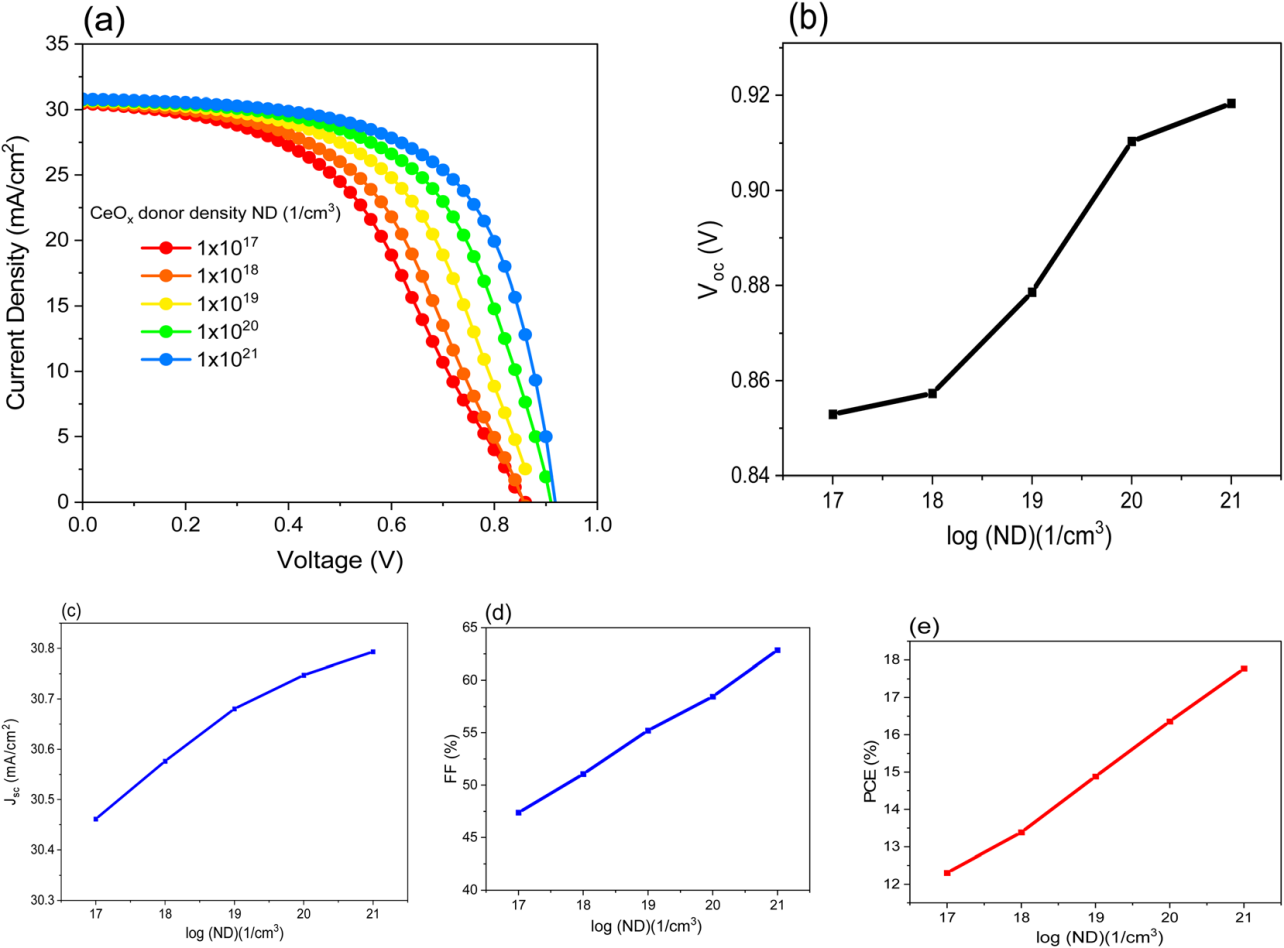
Shows the effect of the ND of CeO_*x*_ on performance of FTO/CeO_*x*_/MASnI_3_/Spiro-OMeTAD/Au PSC where: (a) (*J*–*V*) curve, (b), (c) *J*_SC_, (d) FF and (e) PCE.

The percentage of oxygen content in CeO_*x*_ determines the value of ND.^45^ In this simulation, different values of ND are chosen in order to understand their effect on the PSC performance. Fig. 3a–e shows the variation of the solar cell parameters as a function of log(ND). All the parameter values increase as the value of ND increases. This is attributed to the enhancement of the conductivity, which makes the movement of the electrons through the ETL much easier. ETL's primary function is to give electrons a low-resistive route so that they can be collected. Additionally, it must prevent electrons from stacking up close to the interface. On the other hand, if this happens because of lower conductivity, unrestricted electrons and holes interact together on the interfacial side, resulting in a decreased generated current. Low conductivity causes a higher series resistance (*R*_s_). The high value of resistance in the cell causes the FF to drop. The influence of low conductivity in this case caused by low ND will affect both the FF and *V*_OC_, as shown in Fig. 3b and d, while the *J*_SC_ is not significantly influenced. Table 4 summarizes the obtained results when varying the ND value. It is obvious that the best PCE obtained of 17.77% at ND is equal to 1 × 10^21^ cm^−3^. As the ND of CeO_*x*_ increases to a certain point, the PCE also increases. After this point, the ND of the cerium oxide has no effect on the PCE because the CeO_*x*_ has degenerated (practically). Furthermore, once the ND reaches a certain value where the CeO_*x*_ serves as a hole-blocking and electron-transporting layer, any further increase in the ND value is unnecessary.^46^

**Table 4:** Parameters of FTO/CeO_*x*_/MASnI_3_/Spiro-OMeTAD/Au PSC with differt values of ND of CeO_*x*_

ND (cm^−3^)	*V*_OC_ (V)	*J*_SC_ (mA cm^−2^)	FF (%)	PCE (%)	*V*_m_ (V)	*J*_m_ (mA cm^−2^)
1 × 10^17^	0.85	30.46	47.37	11.82	0.52	23.58
1 × 10^18^	0.86	30.58	51.07	13.37	0.56	24.05
1 × 10^19^	0.88	30.68	55.21	14.87	0.61	24.49
1 × 10^20^	0.91	30.75	58.45	16.35	0.66	24.83
1 × 10^21^	0.92	30.79	62.86	17.77	0.71	25.13

### 3.3 Effect of the series and shunt resistance (*R*_s_ & *R*_sh_)

**Fig. 4:**
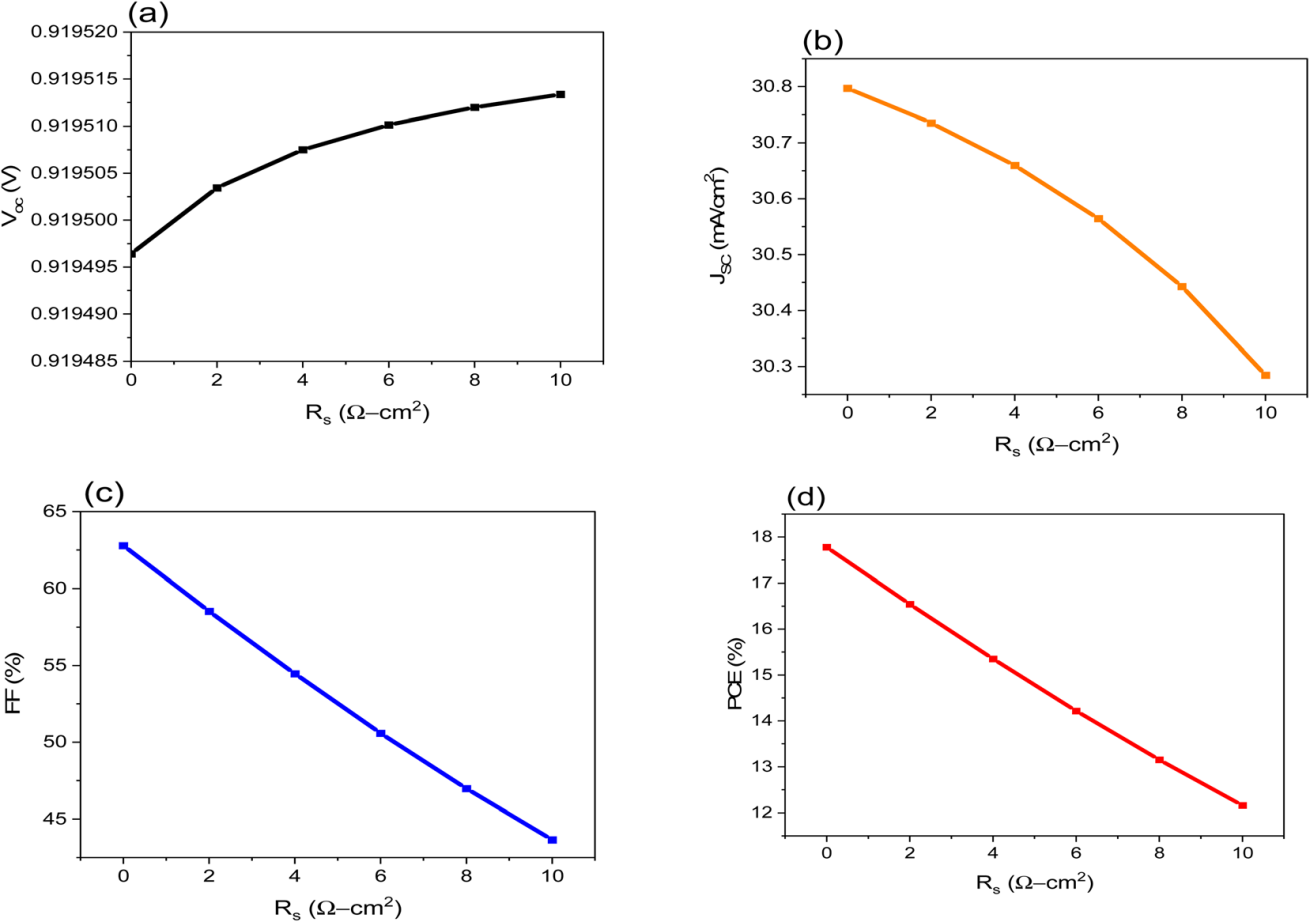
Shows the influence of *R*_s_ on the performance of FTO/CeO_*x*_/MASnI_3_/Spiro-OMeTAD/Au PSC where: (a) *V*_OC_, (b), *J*_SC_, (c) FF and (d) PCE.

The *R*_s_ and *R*_sh_ have significant control over how well solar cells work. It comes from the metal connections on the solar cell and layer surfaces. The device's efficiency was assessed by changing the value of *R*_s_ from 0 to 10 (Ω cm^2^). Fig. 4a–d show that as *R*_s_ increases, the FF and PCE decrease, resulting in leakage currents, while the *V*_OC_ and *J*_SC_ remain unchanged.

**Fig. 5:**
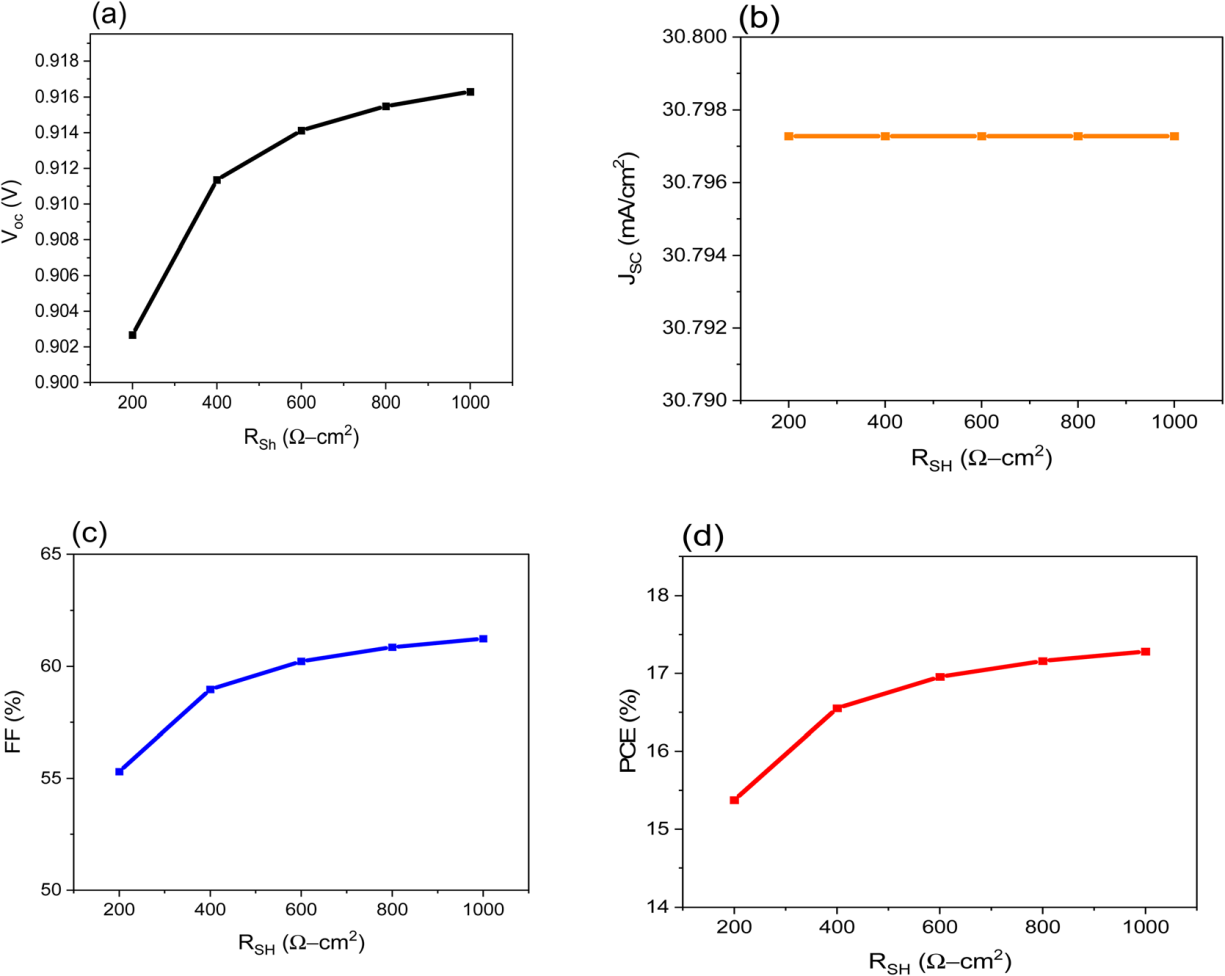
Shows the impact of *R*_sh_ of the on performance of FTO/CeO_*x*_/MASnI_3_/Spiro-OMeTAD/Au PSC where: (a) *V*_OC_, (b), *J*_SC_, (c) FF and (d) PCE.

Poor shunt resistance increases power loss in the solar cell by enabling the current produced by light to take an alternative path. A similar deflection drops the voltage produced by the photovoltaic cell and minimizes the current flowing through the photovoltaic junction. Fig. 5a–d shows that the FF is the most affected parameter, resulting in a decrease in the value of PCE, while the *V*_OC_ and *J*_SC_ are almost unchanged.

Notably, the results showed that PSCs designed with a room-temperature CeO_*x*_ layer could improve the PCE of MASnI_3_-based photovoltaics, which is a significant step toward PSC industrialization. Table 5 shows the results of a comparison between the various structures, and it can be stated that adding CeO_*x*_ into the perovskite-based devices is the most beneficial method for using it in PSCs.

**Table 5:** A comparison between our work and previously published studies used CeO_*x*_ As an ETL

Author	Structure	Perovskite thickness (nm)	Total defect density (cm^−3^)	PCE (%)
Anu *et al.*^47^	FTO/CeO_*x*_/PCBM/MAPbI_3_/Spiro-OMeTAD/Au	250	5 × 10^14^	18.36
Pandey *et al.*^45^	FTO/CeO_*x*_/PCBM/MAPbI_3_/CNTs/Spiro-OMeTAD/Ag	250	2.14 × 10^17^	18.20
Raoui *et al.*^48^	FTO/CeO_*x*_/PCBM/CsSn_0.5_Ge_0.5_I_3_/PTAA/Spiro-OMeTAD/Au	200	1 × 10^11^	24.20
Ahmmed *et al.*^49^	ITO/CeO_*x*_/CsBi_3_I_10_/NiO_*x*_/Au	1000	1 × 10^14^	19.16
Moiz *et al.*^50^	FTO/CeO_*x*_/Cs_2_TiBr_6_/*N*,*N*′-bis(naphthalen-1-yl)-*N*,*N*′-bis(phenyl)-benzidine/Au	150	1 × 10^15^	20.40
This work	FTO/CeO_*x*_/MASnI_3_/Spiro-OMeTAD/Au	600	4.5 × 10^16^	17.77

## 4. Conclusions

The performance of the suggested cell structure FTO/CeO_*x*_/MASnI_3_/Spiro-OMeTAD/Au PSC has been examined using the SCAPS 1D simulation software. Different CeO_*x*_ and MASnI_3_ parameters are tuned in order to investigate the behavior of the device. The open-circuit voltage and efficiency received a significant contribution from the CeO_*x*_ layer. Cell performance was shown to be enhanced by the CeO_*x*_ ETL's higher ND. Cell performance appears to be significantly influenced by the MASnI_3_ layer's thickness. The density of the MASnI_3_/CeO_*x*_/MASnI_3_ interfacial defect and the MASnI_3_/Spiro-OMeTAD interfacial defect were determined. The highest PCE ever calculated was 17.77%, with a *J*_SC_ value of 30.79 mA cm^−2^, a *V*_OC_ value of 0.919 V, and a fill factor of 62.78%. Our findings indicate that future research may show that the suggested device structure involving both CeO_*x*_ and MASnI_3_ is an effective device for making thin-film solar cells that are both inexpensive and efficient.

## Data availability

Data will be available based on reasonable request.

## Ethics approval and consent to participate

We comply with the ethical standards. We provide our consent to take part.

## Conflicts of interest

There are no conflicts to declare.

## Supplementary Material
